# Combination of a hypomethylating agent and inhibitors of PARP and HDAC traps PARP1 and DNMT1 to chromatin, acetylates DNA repair proteins, down-regulates NuRD and induces apoptosis in human leukemia and lymphoma cells

**DOI:** 10.18632/oncotarget.23386

**Published:** 2017-12-17

**Authors:** Benigno C. Valdez, Yang Li, David Murray, Yan Liu, Yago Nieto, Richard E. Champlin, Borje S. Andersson

**Affiliations:** ^1^ Department of Stem Cell Transplantation and Cellular Therapy, The University of Texas MD Anderson Cancer Center, Houston, Texas 77030, USA; ^2^ Department of Experimental Oncology, Cross Cancer Institute, Edmonton, Alberta T6G 1Z2, Canada

**Keywords:** niraparib, olaparib, decitabine, romidepsin, panobinostat

## Abstract

Combination of drugs that target different aspects of aberrant cellular processes is an efficacious treatment for hematological malignancies. Hypomethylating agents (HMAs) and inhibitors of poly(ADP-ribose) polymerases (PARPis) and histone deacetylases (HDACis) are clinically active anti-tumor drugs. We hypothesized that their combination would be synergistically cytotoxic to leukemia and lymphoma cells. Exposure of AML and lymphoma cell lines to the combination of the PARPi niraparib (Npb), the HMA decitabine (DAC) and the HDACi romidepsin (Rom) or panobinostat (Pano) synergistically inhibited cell proliferation by up to 70% via activation of the ATM pathway, increased production of reactive oxygen species, decreased mitochondrial membrane potential, and activated apoptosis. Addition of the DNA alkylating agents busulfan (Bu) and/or melphalan enhanced the anti-proliferative/cytotoxic effects of the triple-drug combination. [Npb+DAC+Rom] significantly increased the level of chromatin-bound PARP1 and DNMT1 and caused acetylation of DNA repair proteins, including Ku70, Ku80, PARP1, DDB1, ERCC1 and XPF/ERCC4. This three-drug combination down-regulated the components of the nucleosome-remodeling deacetylase (NuRD) complex, which is involved in DNA-damage repair. Addition of Bu to this combination further enhanced these effects on NuRD. The trapping of PARP1 and DNMT1 to chromatin, acetylation of DNA repair proteins, and down-regulation of NuRD may all have increased double-strand DNA break (DSB) formation as suggested by activation of the DNA-damage response, concomitantly resulting in tumor cell death. Similar synergistic cytotoxicity was observed in blood mononuclear cells isolated from patients with AML and lymphoma. Our results provide a rationale for the development of [Npb+DAC+Rom/Pano] combination therapies for leukemia and lymphoma patients.

## INTRODUCTION

Most hematological malignancies are caused by defects in multiple cellular events, hence, combination therapies are expected to be more efficacious than single-drug treatments. These cellular abnormalities may be due to aberrant epigenetic changes and/or genetic mutations that alter gene expression. DNA methylation and histone modifications are epigenetic processes whose interplay dictates whether a gene or set of genes is transcriptionally expressed or silenced. Hypermethylation of the CpG islands in the gene promoter regions [1] and deacetylation of histone tails [2], which regulate chromatin conformation, may down-regulate the expression of tumor suppressor genes. The status of DNA methylation and histone acetylation may be modified by hypomethylating agents (HMAs) and histone deacetylase inhibitors (HDACis). The efficacy of these drugs in controlling tumor cell proliferation, individually or in combination, has been shown in pre-clinical models and in clinical trials [3, 4].

In the search for more efficacious drug combinations involving other cellular targets, HMAs have been shown to synergize with inhibitors of poly(ADP-ribose) polymerase (PARPis). HMAs inhibit DNA methylation and induce DNA damage by inactivating and trapping DNA methyltransferase (DNMT) to DNA, with the damaged DNA being repaired by the base excision repair (BER) machinery [5–7]. Since olaparib, a PARPi, disrupts the repair of HMA-induced DNMT1-DNA lesions by preventing relocation of the BER enzyme XRCC1 to DNA damage sites, the combination of olaparib with the HMA decitabine (DAC) provided synergistic cytotoxicity [7]. This synergism is also attributed to PARPi-mediated trapping of PARP to DNA [8] as shown by the combination of the PARPi talazoparib and DAC which trap both PARP1 and DNMT1 to DNA, resulting in increased levels of double-strand DNA breaks (DSBs) [9].

PARPis are also synergistic with HDACis. Exposure of leukemic cell lines to the PARPi P10 and the HDACi SAHA induced S phase arrest due to increased DNA damage and replicative stress [10]. The HDACi trichostatin A increased acetylation of DNA repair factors and impaired the non-homologous end joining (NHEJ) DNA repair pathway, and addition of talazoparib enhanced trapping of PARP1 to DSBs leading to decreased NHEJ and leukemia cell death [11].

These reported synergistic cytotoxicities of PARPis with either HMAs or HDACis suggest that combination of the three types of drugs may result in much greater cell death. We, therefore, determined their activity in both leukemia and lymphoma cell lines and patient cell samples. Our study shows significant synergism when the three groups of drugs were combined. Addition of a DNA alkylating agent further increased their anti-proliferative and pro-apoptotic activity. Our results provide a pre-clinical rationale for combined administration of these drugs in forthcoming clinical trials.

## RESULTS

### Combination of HMA, PARPi and HDACi provides synergistic cytotoxicity towards leukemia and lymphoma cell lines

**Figure 1:**
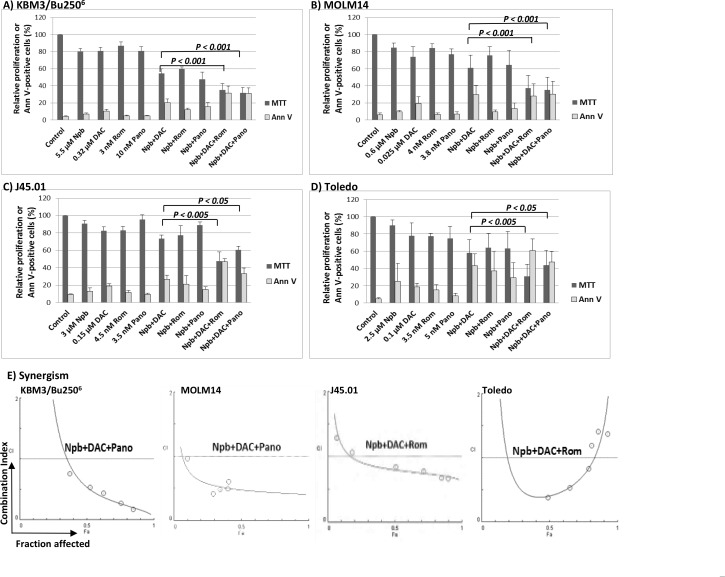
Synergistic anti-proliferative and cytotoxic effects of the various drug combinations in leukemia **(A**, **B**) and lymphoma (**C**, **D**) cell lines. Cells were exposed to drugs, alone or in combination, for 48 hrs then analyzed for cell proliferation by MTT assay and for apoptosis by Annexin V (Ann V) assay. Results are average ± SD of at least three independent experiments. Statistically significant differences are indicated by *P* values. The relationships between combination index (CI; y-axis) and fraction affected (Fa; x-axis) for the MTT assay data are shown in panel (**E**). The graphs are representatives of two independent experiments. CI < 1 indicates synergism. Npb, niraparib; Ola, olaparib; DAC, decitabine; Rom, romidepsin; Pano, panobinostat.

Exposure of KBM3/Bu250^6^ and MOLM14 cell lines (both AML) to concentrations close to IC_20_ values (the concentration of drug required for 20% growth inhibition) of the PARPi niraparib (Npb), the hypomethylating agent DAC, or the HDACi romidepsin (Rom) or panobinostat (Pano) inhibited cell proliferation by ∼20%, as expected, when administered individually, as measured by the MTT assay (Figure 1A, 1B). Using the same drug concentrations, the two-drug combinations [Npb+DAC], [Npb+Rom] and [Npb+Pano] decreased KBM3/Bu250^6^ proliferation to ∼54%, ∼60%, and ∼48%, respectively, versus untreated control cells (Figure 1A). The same two-drug combinations decreased the proliferation of MOLM14 cells to ∼61%, ∼76% and ∼64% of control, respectively (Figure 1B). Addition of the HDACi to [Npb+DAC] significantly decreased proliferation of KBM3/Bu250^6^ cells to ∼35% (with Rom, *P* < 0.001) and ∼32% (with Pano, *P* < 0.001) of control levels while exposure of MOLM14 to [Npb+DAC+Rom] or [Npb+DAC+Pano] resulted in ∼42% (*P* < 0.001) and ∼39% (*P* < 0.001) of control proliferation, respectively.

A similar MTT assay for cell proliferation was performed using two lymphoma model cell lines, J45.01 (T lymphoma cell line) and Toledo (B lymphoma cell line). Using drug concentrations close to their IC_20_ values, exposure of J45.01 cells to [Npb+DAC], [Npb+Rom] and [Npb+Pano] combinations resulted in cell proliferation of ∼73%, ∼77% and ∼89% of control, respectively. Addition of Rom or Pano to [Npb+DAC] resulted in ∼48% (*P* < 0.005) and ∼61% (*P* < 0.05) proliferation versus control, respectively (Figure 1C). Exposure of Toledo cells to [Npb+DAC], [Npb+Rom] and [Npb+Pano] combinations resulted in cell proliferation of ∼58%, ∼64% and ∼63%, respectively, compared to control. The anti-proliferative effects of [Npb+DAC] significantly increased when Rom and Pano were added, resulting in ∼31% (*P* < 0.005) and ∼44% (*P* < 0.05) proliferation versus control, respectively (Figure 1D).

To test for synergistic interactions, cells were exposed to different concentrations of individual drugs or to the three-drug combinations at a constant concentration ratio, and the MTT assay was performed after 48 hrs. The calculated combination index (CI) values at increasing drug effects were graphically analyzed and shown in Figure 1E for each cell line as indicated. The calculated CI values less than 1 suggest significant synergism in the four cell lines.

The observed synergistic inhibition of cellular proliferation by [Npb+DAC+Rom/Pano] correlates with the activation of apoptosis as determined by Annexin V assay (Figure 1). Exposure of the four cell lines to the three-drug combinations resulted in ∼25%–61% Annexin V-positive cells whereas the individual drugs and other combinations showed much lesser effects. Overall, these results suggest strong synergistic cytotoxicity of Npb, DAC and Rom/Pano in leukemia and lymphoma cell lines.

### [Npb+DAC+Rom/Pano] combination activates the DNA-damage response and apoptosis pathways

**Figure 2:**
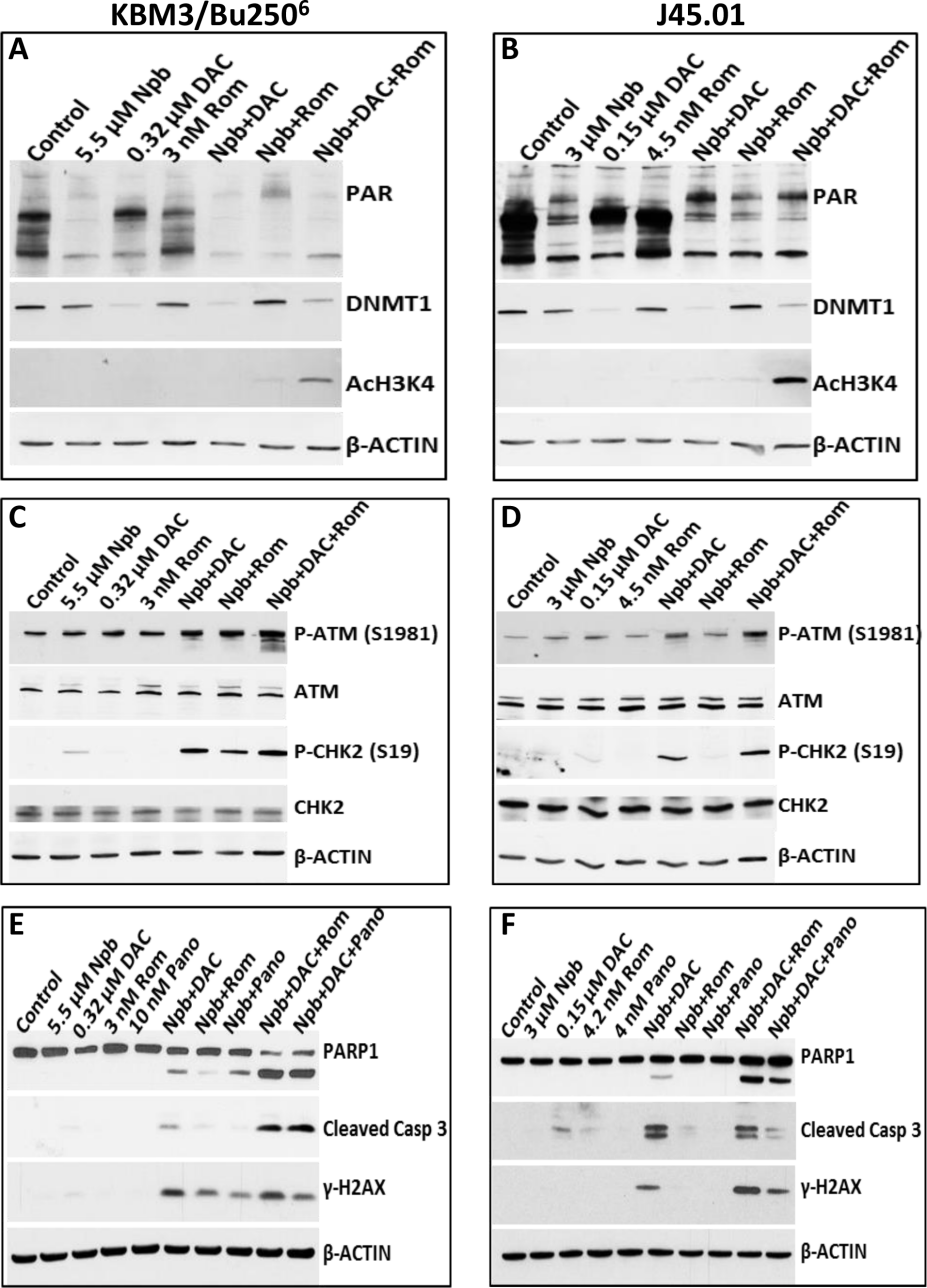
Analysis of drug targets (**A**, **B**) and activation of the ATM pathway (**C**, **D**) and apoptosis (**E**, **F**). Cells were exposed to drugs, alone or in combination, for 48 hrs and total cell extracts were analyzed by Western blotting using antibodies to the indicated proteins. Results are representatives of two independent experiments. Npb, niraparib; DAC, decitabine; Rom, romidepsin; Pano, panobinostat.

To determine possible mechanisms of the observed synergistic cytotoxicity, we initially sought to analyze the target molecules of each drug. Exposure of KBM3/Bu250^6^ and J45.01 cells to Npb, alone or in combination with other drugs, decreased the levels of poly-ADP ribosylated (PAR) proteins whereas DAC and Rom had insignificant effects thereon (Figure 2A, 2B). DAC, but not Rom, decreased the level of DNMT1, as expected [12]; Npb slightly decreased DNMT1 expression (Figure 2A, 2B). Of the various treatment groups, only the combination of Rom with Npb and DAC induced acetylation of histone 3 at lysine 4 (Figure 2A, 2B); the lack of effect of Rom alone may be due to the relatively low drug concentration, since we previously showed that a higher concentration of Rom (10 μM) did cause significant acetylation of histone 3 [13]. These results suggest that Npb, DAC and Rom do affect their respective target molecules in our cell line models.

Since PARPi and HMA may cause DNA damage [14, 15], we examined if [Npb+DAC+Rom] would activate the DNA-damage response (DDR). Exposure of KBM3/Bu250^6^ and J45.01 cells to the three-drug combination dramatically increased the phosphorylation of ATM kinase at Ser1981 (Figure 2C, 2D) and of two of its known substrates, CHK2 and H2AX (Figure 2C–2F), suggesting activation of the DDR pathway.

Whether the observed DDR might lead to apoptosis was assessed by analyzing the cleavage of PARP1 and CASPASE 3. Significant protein cleavage was observed in cells exposed to [Npb+DAC+Rom/Pano] (Figure 2E, 2F), suggesting activation of apoptosis consistent with the observed increase in Annexin V-positive cells (Figure 1).

### [Npb+DAC+Rom] traps PARP1 and DNMT1 to chromatin

**Figure 3:**
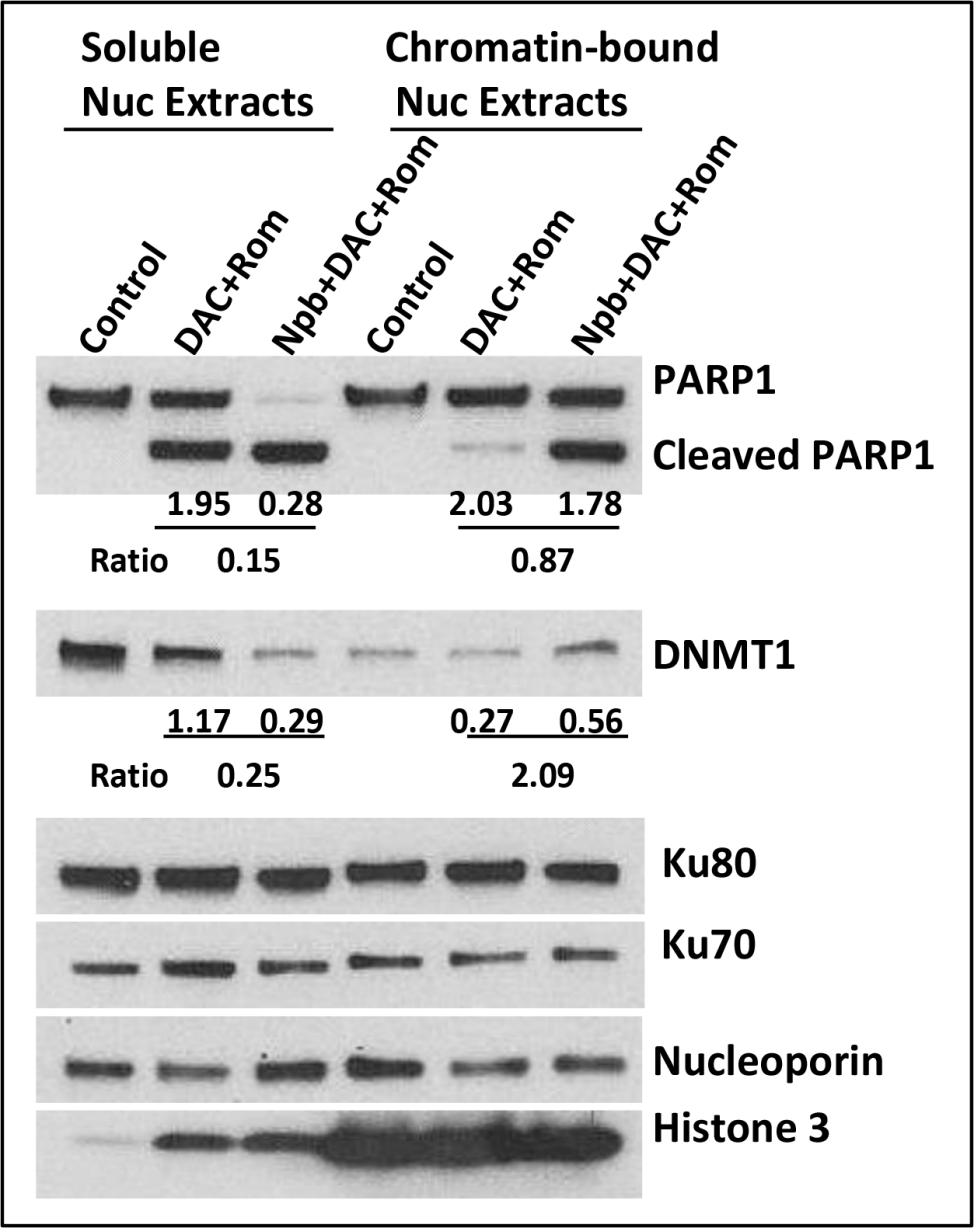
Drug-mediated trapping of PARP1 and DNMT1 to DNA J45.01 cells were exposed to the indicated drug(s) for 48 hrs and cell fractions were isolated as described under Materials and Methods. Soluble and chromatin-bound nuclear proteins were analyzed by Western blotting. The blot signals for uncleaved PARP1 and DNMT1 were normalized relative to nucleoporin and the ratio between [DAC+Rom+Npb]/[DAC+Rom] was calculated. Results are representatives of two independent experiments. Nuc, nuclear; Npb, niraparib; DAC, decitabine; Rom, romidepsin.

In search of other molecular mechanisms underlying the observed drug-mediated cell death, we examined the effects of [DAC+Rom±Npb] on the trapping of PARP1 and DNMT1 to chromatin. Recent reports showed that PARPi and HMA trapped PARP1 and DNMT1 on damaged DNA sites [9] and that HDACi trapped PARP1 on DSBs [11]. J45.01 cells were therefore exposed to drugs for 48 hrs and cellular fractions were isolated. Soluble and chromatin-bound nuclear proteins were analyzed by Western blotting. Consistent with Figure 2, two- and three-drug combinations caused cleavage of PARP1 (Figure 3). However, only the signals for uncleaved PARP1 were quantitatively analyzed (Figure 3). The soluble nuclear extracts showed less uncleaved PARP1 in cells exposed to [DAC+Rom+Npb] than [DAC+Rom] (ratio of 0.15:1) while the chromatin-bound fractions showed a similar level of uncleaved PARP1 (ratio of 0.87:1), suggesting that addition of Npb to [DAC+Rom] increased the binding or trapping of PARP1 to chromatin. Similar analysis for DNMT1 showed increased levels of DNMT1 in the chromatin-bound fraction from cells exposed to [DAC+Rom+Npb] compared with cells exposed to [DAC+Rom]; the DNMT1 ratio was 0.25:1 ([DAC+Rom+Npb]:[DAC+Rom]) in the soluble extracts and 2.09:1 in the chromatin-bound fractions (Figure 3). These results suggest increased DNMT1 trapping to chromatin in the presence of [DAC+Rom+Npb]. Other proteins known to bind to DNA, including Ku80 and Ku70, were not significantly affected. The presence of histone 3 in the chromatin-bound fraction shows the quality of its preparation.

### [Npb+Rom±DAC] increases the acetylation of DNA repair proteins

**Figure 4:**
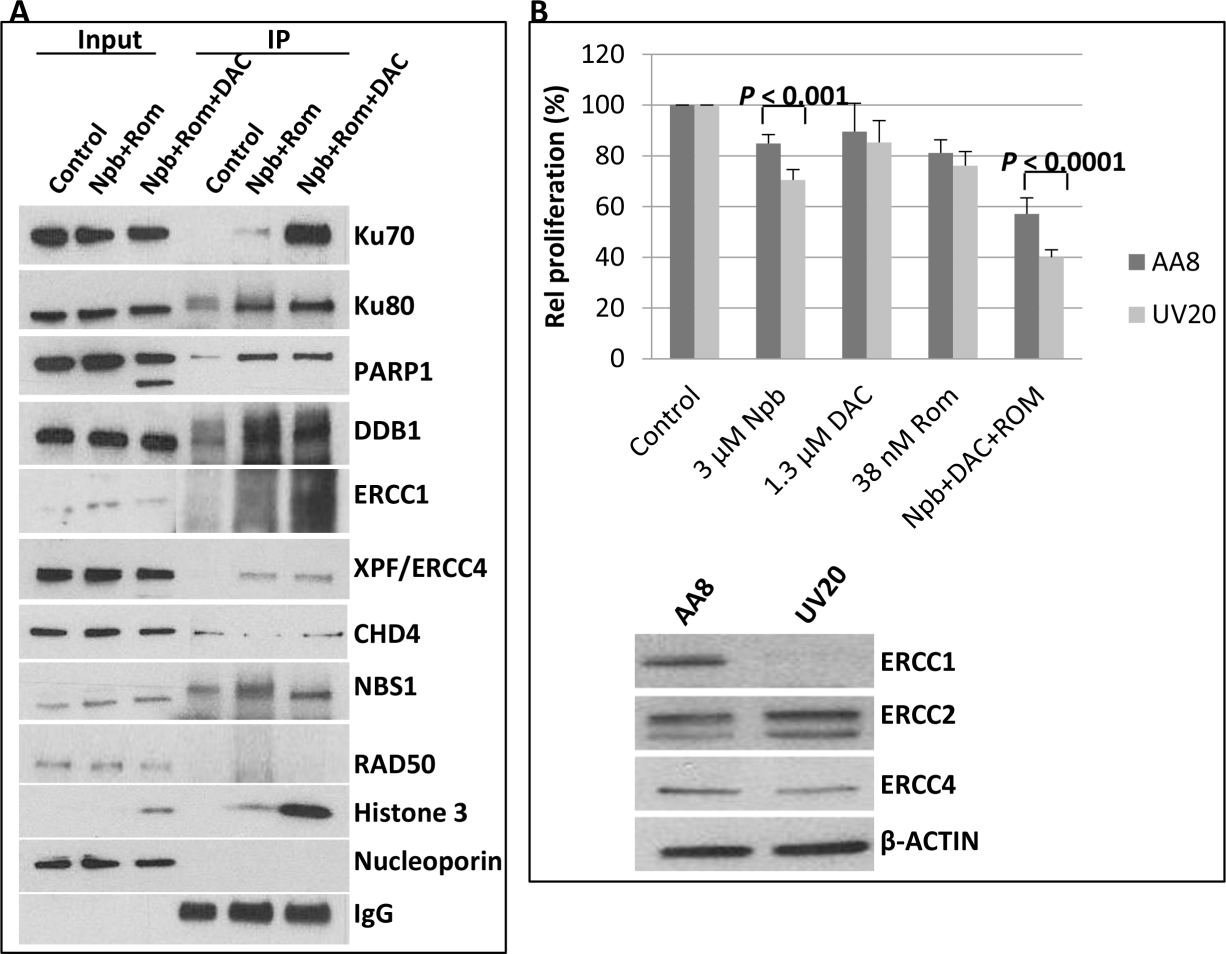
Immunoprecipitation of acetylated proteins and drug sensitivity of ERCC1-deficient cells (**A**) J45.01 cells were exposed to drugs for 48 hrs and acetylated proteins in the nuclear extracts were immunoprecipitated using anti-acetylated lysine antibody as described under Materials and Methods. Approximately 5% of the input for immunoprecipitation was used for Western blot analysis. (**B**) Chinese hamster ovary cell lines AA8 (wild type) and UV20 (ERCC1-deficient) were exposed to the indicated drugs for 48 hrs and analyzed for proliferation by MTT assay (upper panel). Untreated cells were analyzed by Western blotting to show differences in expression of DNA repair proteins (lower panel). Results are representatives of two independent experiments. IP, immunoprecipitate; Npb, niraparib; DAC, decitabine; Rom, romidepsin.

The observed trapping of PARP1 and DNMT1 to chromatin could be a contributing factor for increased DSBs. Another contributing factor might be the acetylation of DNA repair proteins, as previously reported [11]. To examine this possibility, cells were exposed to drugs for 48 hrs and nuclear fractions were isolated. Acetylated proteins were immunoprecipitated using anti-acetylated lysine antibody and analyzed by Western blotting using antibodies against the proteins of interest. The input or starting fraction was included to show presence of the protein. Analysis of the immunoprecipitated acetylated proteins showed increased levels of acetylated Ku70, Ku80, PARP1, DDB1, ERCC1 and XPF/ERCC4, which are DNA repair proteins [16–18], in cells exposed to [Npb+ Rom+DAC] (Figure 4). CHD4 and NBS1 seemed to be constitutively acetylated, while no acetylated RAD50 was immunoprecipitated. The acetylation of histone 3, shown in Figure 2B, was used as a positive control in this experiment (Figure 4). The acetylation sites in some of these proteins, identified by peptide sequencing of acetylomes, are shown in Supplementary Table 1. The level of IgG shows almost equal loading when probed with anti-acetylated lysine antibody. Knowing that acetylation inhibits DNA repair [11], these results suggest that the synergistic cytotoxicity of [Npb+Rom+DAC] is partly due to inhibition of DNA repair through increased protein acetylation.

A possible mechanism that may have contributed to the synergy of the [Npb+DAC+Rom] combination is that the observed inhibition of DNA repair might result in a synthetic-lethal interaction whereby cells with certain DNA repair deficiencies (in this case caused by the drug treatment) are sensitive to PARPis [20]. An example is the known sensitivity of ERCC1-deficient lung cancer cells to PARPis such as Npb [21]. To see if such an effect might be operating with these drug combinations, which clearly have an impact on the DNA repair machinery, we exposed the Chinese hamster ovary (CHO) cell line UV20, which is deficient of nucleotide excision repair protein ERCC1 [19] (Figure 4B), to the 3 drugs, alone or in combination, and analyzed proliferation after 48 hrs using the MTT assay. UV20 cells were noticeably more sensitive to the PARPi Npb (*P* < 0.001) and to the [Npb+DAC+Rom] combination (*P* < 0.0001) compared with the wild-type AA8 cells (Figure 4B), supporting the potential role of synthetic lethality under these conditions.

### A bifunctional DNA alkylator enhances the cytotoxicity of [Npb+DAC+Rom/Pano]

**Figure 5:**
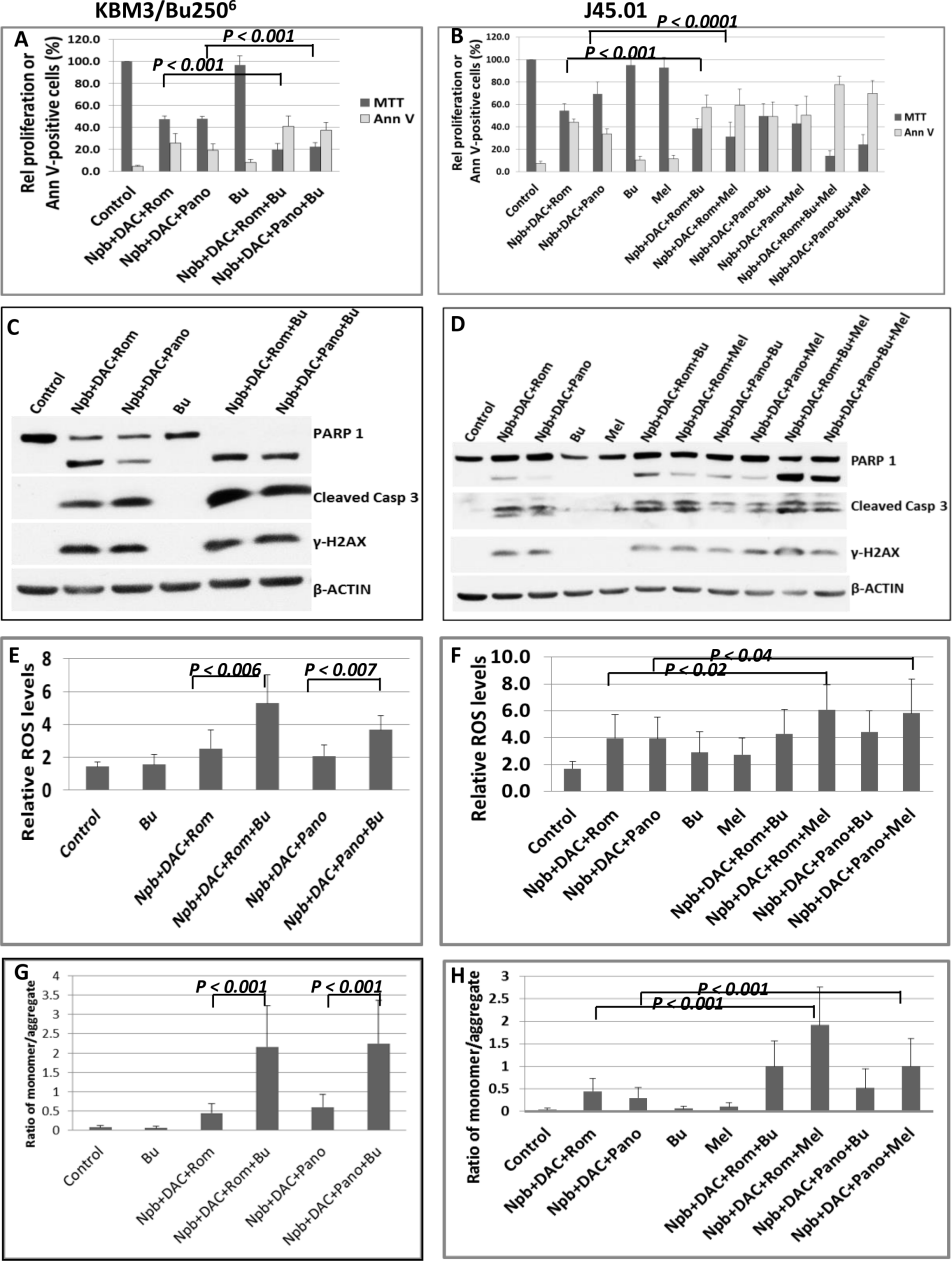
Effects of PARPi, DAC, HDACi, and DNA alkylators on KBM3/Bu250^6^ and J45.01 cells Cells were exposed to drugs, alone or in combination, for 48 hrs and analyzed for proliferation and cell death (**A** and **B**), apoptosis markers by Western blotting (**C** and **D**), ROS production (**E**, **F**) and changes in mitochondrial membrane potential (**G**, **H**). Results in panels A, B, and E–H are average ± SD of at least three independent experiments. Statistically significant differences are indicated by *P* values. Results in C and D are representatives of two independent experiments. Drug abbreviations and concentrations are the same as those shown in Figure 2 except for the inclusion of Bu, busulfan (48 μM for KBM3/Bu250^6^ and 28 μM for J45.01); and/or Mel, melphalan (0.6 μM).

DNA alkylators are common components of pre-transplant conditioning regimens for leukemia and lymphoma patients [22]. Epigenetic modifiers are known to improve their efficacy [22–25]. We therefore sought to determine if addition of a DNA alkylating agent to [Npb+DAC+Rom/Pano] would further improve its anti-proliferative/cytotoxic activity. Addition of busulfan (Bu) to this drug combination decreased proliferation of the AML cell line KBM3/Bu250^6^ from ∼48% to ∼20% of control (Figure 5A). Addition of Bu or melphalan (Mel) to [Npb+DAC+Rom] decreased proliferation of J45.01 cells from ∼53% to ∼38% or ∼30%, respectively, and addition of [Bu+Mel] further decreased proliferation to ∼14% (Figure 5B). Similar results were obtained when Bu or Mel, or both, was/were added to [Npb+DAC+Pano]. The proliferation of J45.01 cells decreased from ∼68% to ∼50%, ∼42%, or ∼23% when Bu or Mel, or both, was/were added to the Pano-containing combination (Figure 5B). These enhanced inhibitory effects on cell proliferation were accompanied by correspondingly increased numbers of Annexin V-positive cells (Figure 5A, 5B) as well as increased cleavage of PARP1 and CASPASE 3 (Figure 5C, 5D), suggesting increased apoptosis.

To identify possible mechanisms of apoptosis activation, we examined the production of reactive oxygen species (ROS) in these cell lines. [Npb+DAC+Rom/Pano+Bu] increased the level of ROS in KBM3/Bu250^6^ cells (Figure 5E); [Npb+DAC+Rom+Bu/Mel] showed a similar effect on ROS production in J45.01 cells (Figure 5F). Since ROS may damage mitochondrial membranes and cause leakage of proapoptotic factors that normally reside in the mitochondria [25], we determined changes in the mitochondrial membrane potential in cells exposed to individual or combined drugs using the JC-1 assay by flow cytometry. JC-1 aggregates stay in the mitochondria while its monomeric form localizes to the cytoplasm. Damage to the mitochondrial membrane causes JC-1 to leak out of the mitochondria. A high ratio of JC-1 monomer/aggregate form indicates a decreased membrane potential. Increased monomer/aggregate ratios were apparent in the presence of [Npb+DAC+Rom/Pano+Bu] in KBM3/Bu250^6^ cells (Figure 5G) and of [Npb+DAC+Rom+Bu/Mel] in J45.01 cells (Figure 5H), suggesting decreased mitochondrial membrane potential which might have contributed to the elevated levels of apoptosis seen with addition of the alkylating agent.

### [Npb+DAC+Rom] down regulates NuRD components; Bu enhances this effect

**Figure 6:**
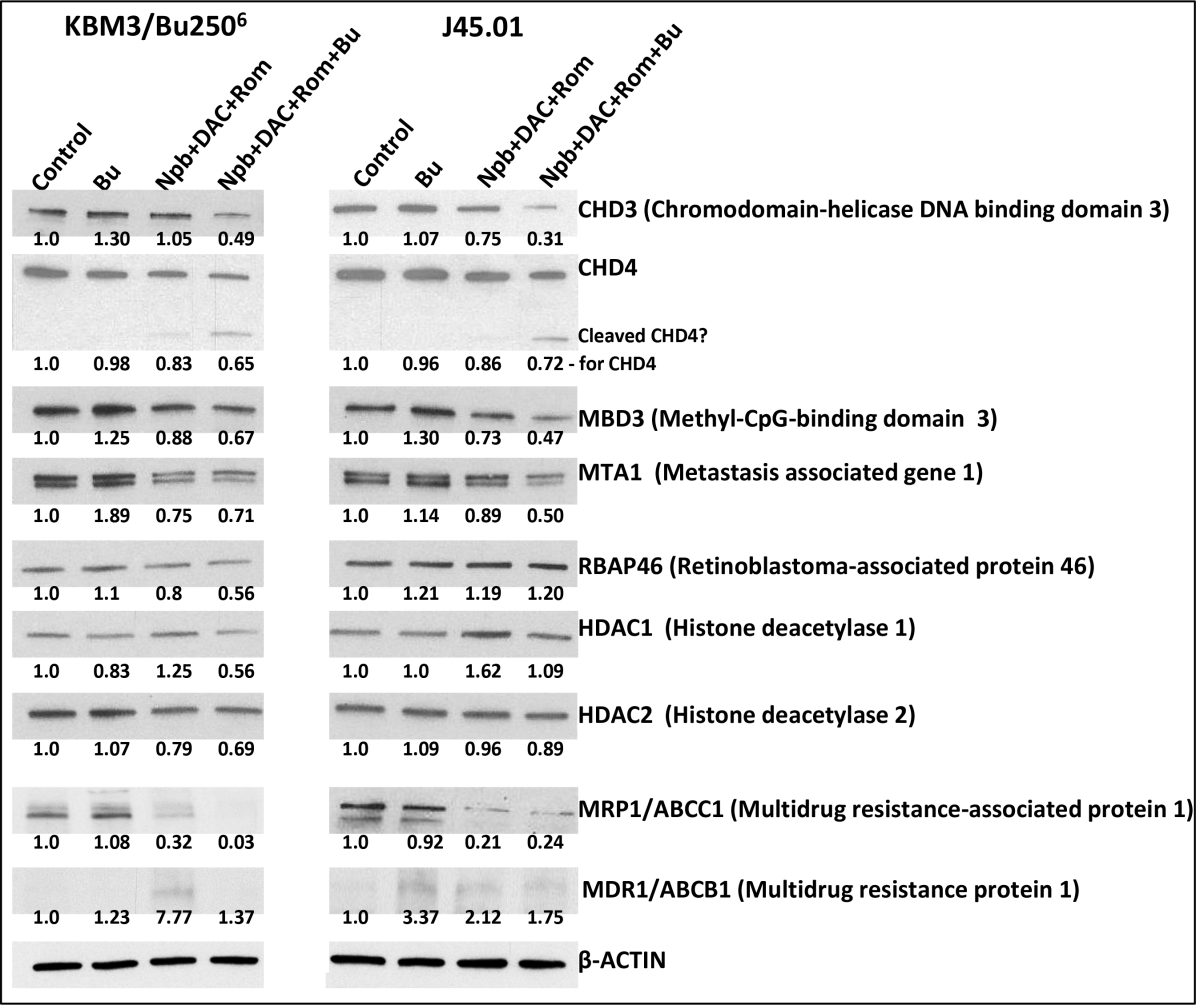
Effects of drug combinations on the NuRD complex components and drug transporters Cells were exposed to the indicated drug(s) for 48 hrs and total cell extracts were analyzed by Western blotting. Drug concentrations are the same as in Figure 5. Results are representatives of two independent experiments. Bu, busulfan; Npb, niraparib; DAC, decitabine; Rom, romidepsin.

The nucleosome remodeling and histone deacetylation complex (NuRD) is a transcription repressor functionally linked to DSB repair [27]. Since PARPi, HMA and HDACi affect cellular processes including chromosome remodeling and DNA repair, we sought to determine the effects of [Npb+DAC+Rom ± Bu] on the components of the NuRD complex. [Npb+DAC+Rom] combination decreased the levels of CHD4, MBD3, MTA1, RBAP46, and HDAC2 to ∼75%–88% of the control in the KBM3/Bu250^6^ cells, and exposure of J45.01 cells to the same drug combination decreased the levels of CHD3, CHD4, MBD3, and MTA1 to ∼73%–89% of the control (Figure 6). Addition of Bu to the three-drug combination in KBM3/Bu250^6^ cells further enhanced these effects; the levels of CHD3, CHD4, MBD3, MTA1, RBAP46, HDAC1 and HDAC2 decreased to ∼49%–71% of the control. Similar effects were observed in J45.01 cells where the Bu-containing combination decreased the levels of CHD3, CHD4, MBD3, MTA1 and HDAC2 to ∼31%–89% of the control (Figure 6). Noticeably, an apparent cleavage of CHD4 occurred in cells exposed to the three-drug combination and was further enhanced in the presence of Bu in both cell lines (Figure 6). These results suggest that [Npb+DAC+Rom ± Bu] mediates the down regulation of NuRD components that may compromise DNA repair and contribute to increased DSB formation which consequently leads to apoptosis.

We previously showed that cellular exposure to HDACi decreased the level of the drug transporter MRP1/ABCC1 but increased the level of MDR1/ABCB1 [28]. Similar effects were observed when KBM3/Bu250^6^ and J45.01 cells were exposed to [Npb+DAC+Rom] (Figure 6). [Npb+DAC+Rom] decreased MRP1 to ∼21%-32% of controls in the two cell lines which further decreased to ∼3% when Bu was added in KBM3/Bu250^6^ cells (Figure 6). Since MRP1/ABCC1 mediates efflux of glutathionylated bifunctional alkylators [29], its down-regulation may consequently lead to retention of more free/active Bu in the cell. The efficacy of [Npb+DAC+Rom+Bu] in decreasing the levels of some NuRD components (Figure 6) and activating apoptosis (Figure 5) might thus be partly due to down-regulation of MRP1/ABCC1, resulting in an increased intracellular concentration of free Bu.

### Exposure of mononuclear cells from patients with leukemia to [Npb+DAC+Rom] or with lymphoma to [Npb+DAC+Rom+Bu+Mel] activates the DNA-damage response and apoptosis

**Figure 7:**
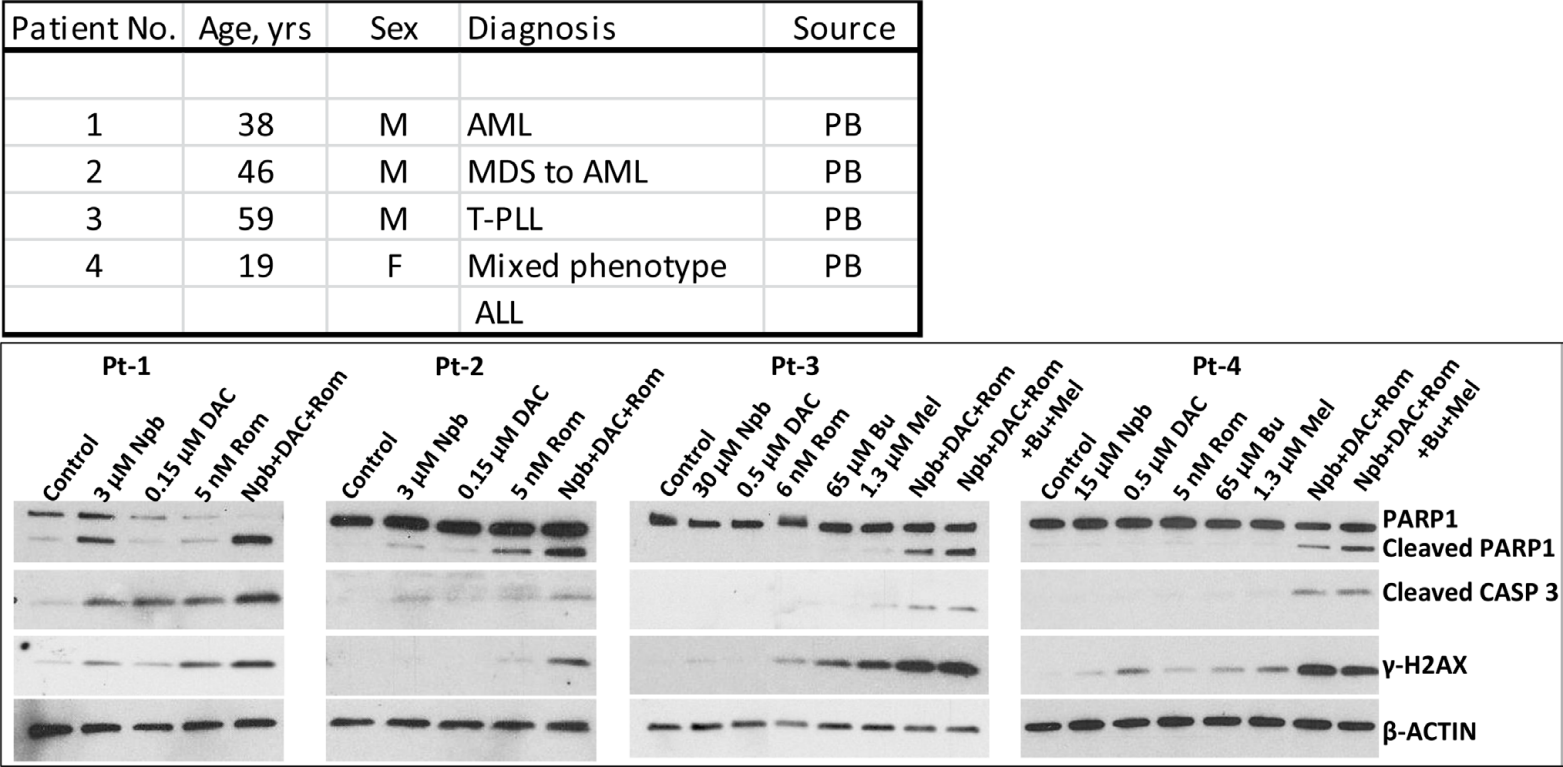
Effect of exposure of patient cell samples to drugs on biomarkers of apoptosis and DNA-damage response Mononuclear cells were isolated from peripheral blood of patients with acute myeloid leukemia (AML), T-cell prolymphocytic leukemia (T-PLL), or acute lymphoblastic leukemia (ALL, mixed phenotype with AML), and exposed to drug(s) for 48 hrs. Total cell extracts were analyzed by Western blotting. PB, peripheral blood; Npb, niraparib; DAC, decitabine; Rom, romidepsin; Bu, busulfan; Mel, melphalan.

To determine the potential clinical significance of our cell line studies, we isolated mononuclear cells from patients with leukemia or lymphoma, exposed them to individual drugs (or combinations) and analyzed for levels of selected proteins by Western blotting. Increased phosphorylation of γ-H2AX was observed in cells from two AML patients exposed to [Npb+DAC+Rom] and in cells from two lymphoma patients exposed to [Npb+DAC+Rom ± (Bu+Mel)], indicative of DDR activation (Figure 7). PARP1 and CASPASE 3 were significantly cleaved in cells exposed to these combinations, suggesting activation of apoptosis. These results show drug synergism in cells derived from patients with leukemia or lymphoma involving mechanisms analogous to those seen in cultured cell lines.

## DISCUSSION

**Figure 8:**
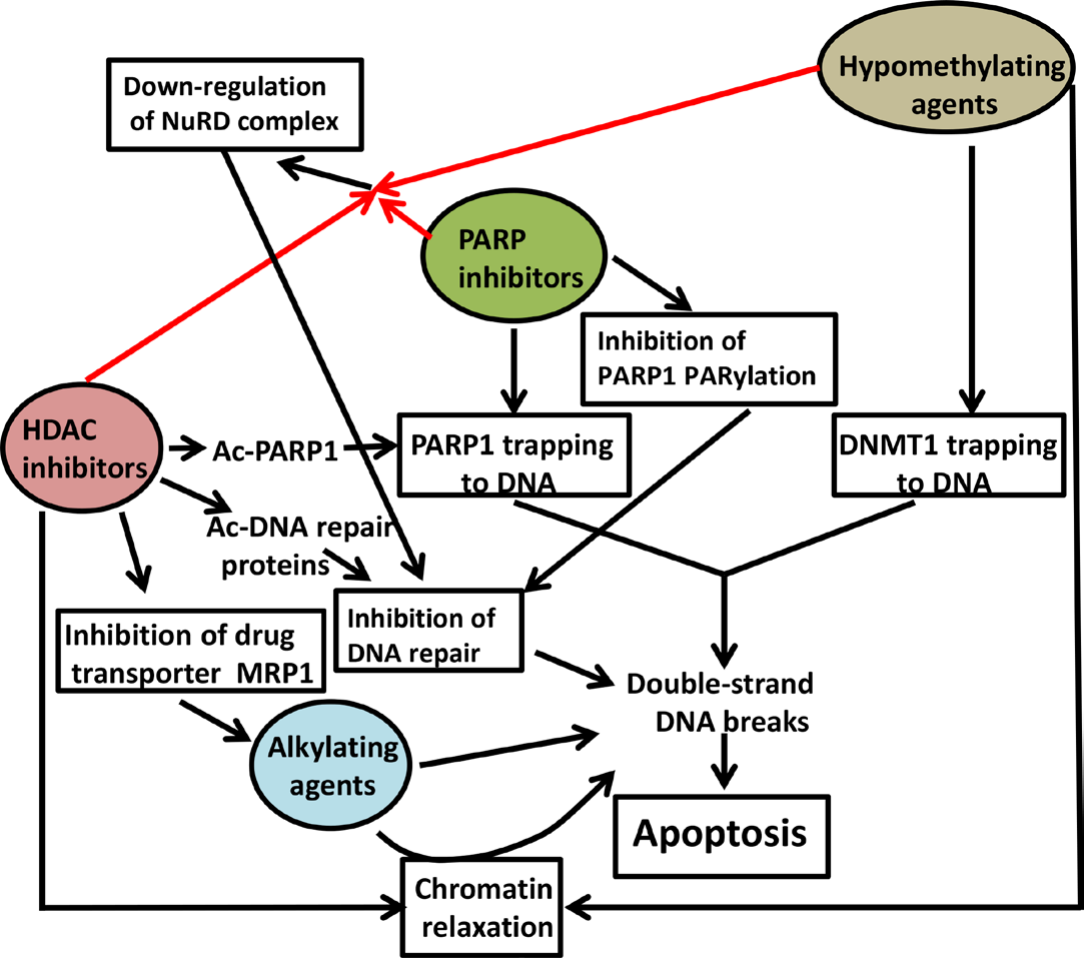
Suggested mechanisms of synergistic cytotoxicity PARP inhibitors (e.g., niraparib) and hypomethylating agents (e.g., decitabine) cause trapping of PARP1 and DNMT1 on DNA, respectively, and increase double-strand DNA breaks (DSBs) during DNA replication. Inhibition of PARP1 self-PARylation results in inefficient recruitment of DNA repair proteins to DSBs. HDAC inhibitors (e.g., romidepsin and panobinostat) increase acetylation of DNA repair proteins including PARP1 and inhibit DNA repair. HDAC inhibitors also decrease the MRP1 transporter, resulting in increased intracellular concentration of alkylating agents and thus an increase in DSBs. HDAC inhibitors and hypomethylating agents also relax chromatin structure and make DNA more susceptible to alkylation. The combination of PARP and HDAC inhibitors and hypomethylating agents down-regulates components of the NuRD complex, resulting in inhibition of DNA repair. The overwhelming increase in DSBs commits cells to apoptosis. Ac, acetyl.

This study presents evidence and possible mechanisms of synergistic cytotoxicity of [Npb+DAC+Rom/Pano] combinations which is further enhanced in the presence of DNA alkylating agents. This synergism was observed in leukemia and lymphoma cell lines, and similar molecular events were seen in mononuclear cells isolated from patients with AML and lymphoid leukemia/lymphoma, suggesting a general cytotoxic efficacy of these combinations. This cytotoxicity may be attributed to the combined effects of the drugs in indirectly inflicting DNA damage, and effectively preventing its repair, which consequently activates the apoptotic pathway (Figure 8).

PARP recognizes single-strand DNA breaks and PARylates itself, resulting in recruitment and PARylation of various DNA repair proteins including XRCC1, DNA-PKcs, Ku70, Ku80, ATM, and others [30]. PARP inhibitors trap PARP to the sites of DNA damage [8]. HDAC inhibitors also cause chromatin-trapping of PARP1 by increasing its acetylated form [11]. On the other hand, hypomethylating agents like DAC cause covalent linkage of DNMT1 to DNA [31]. These PARP1-DNA and DNMT1-DNA complexes inhibit DNA repair, transcription and replication which leads to formation of DSBs and cell death. Our results support and extend these previous studies. The [Npb+DAC+Rom] combination increased trapping of PARP1 and DNMT1 to chromatin (Figure 3). Moreover, the observed increase in acetylation of DNA repair proteins such as Ku70, Ku80, PARP1, DDB1, ERCC1 and XPF/ERCC4 (Figure 4), which may lead to inhibition of DNA repair [11], is consistent with this mechanism. Such inhibition of DNA repair proteins is also consistent with the enhanced synergistic cytotoxicity of [Npb+DAC+Rom] when the DNA alkylating agent Bu was added (Figure 5). This is also consistent with previous reports from our laboratory showing an increased level of sensitivity of ERCC1- and XPF/ERCC4-deficient mouse and human cell lines to DNA alkylating agents, such as activated cyclophosphamide/phosphoramide mustard [19, 32].

Another possible mechanism of their synergistic cytotoxicity is the efficacy of [Npb+DAC+Rom] in down-regulating the components of NuRD (Figure 6), a complex that remodels chromatin to activate DDR and facilitate repair of damaged DNA [33]. NuRD represses transcription to provide appropriate time and binding surfaces for the DNA repair machinery, limit the mobility of the DNA breaks, and maintain the DNA ends in close contact [33]. The repressive chromatin conformation is maintained by the histone deacetylase activities of HDAC1 and HDAC2 [34]. NuRD is directly recruited to the DNA damage sites through binding of its component CHD4 to PARylated proteins [35]. It is possible that inhibition of deacetylation and PARylation of scaffold proteins with Rom and Npb, respectively, may destabilize the complex and lead to degradation of its components. This supposition is consistent with the observed drug-mediated cleavage of CHD4 (Figure 6). Additional studies are needed to determine the actual mechanism of drug-mediated down-regulation of NuRD and how it leads to inhibition of DNA repair and concomitant accumulation of DSBs (Figure 8).

The presence of the DNA alkylator Bu exacerbates the down-regulation of the NuRD complex and further enhances the cytotoxicity of [Npb+DAC+Rom] (Figure 6). The presence of DAC and Rom in the combination may relax chromatin [36] and make the DNA more susceptible to Bu alkylation. Furthermore, the observed down-regulation of the Bu transporter MRP1/ABCC1 may result in an increased intracellular concentration of the active alkylating agent, resulting in more pronounced DNA damage (Figure 8).

These pharmacological effects appear to converge to a common mechanism of increasing DSBs that lead to cell death. The increased phosphorylation of H2AX, an indicator of DDR, suggests increased DSB levels in cells exposed to [Npb+DAC+Rom/Pano] (Figure 2). Activation of the DDR has been shown to induce generation of ROS [37], which damages mitochondrial membranes and causes leakage of pro-apoptotic factors [26]. Our results show increased ROS production and decreased mitochondrial membrane potential in both leukemia and lymphoma cell lines exposed to these drug combinations with a pattern that broadly parallels their effect on cytotoxicity (Figure 5). The synthetic lethality imposed by the [Npb+DAC+Rom] combination in ERCC1-deficient cells (Figure 4B) also supports our model.

In summary, this study provides evidence of interrelated mechanisms that converge to generate a complex genotoxic insult of increased DSBs in both leukemia and lymphoma cell lines and patient-derived samples. These results should be informative for the design of clinical trials to evaluate the efficacy of these drug combinations as components of intensified induction therapy or as part of optimized pre-transplant conditioning regimens for patients with both myeloid and lymphoid malignancies. Such studies will be similar to published clinical trials on the combination of PARP inhibitor velaparib with temozolomide [38] or topotecan and carboplatin [39] in acute leukemias.

## MATERIALS AND METHODS

### Cell culture, patient samples and drugs

KBM3/Bu250^6^ is a busulfan-resistant AML cell line established in our laboratory [40]. MOLM14 is an AML cell line obtained from the laboratory of Dr. Michael Andreeff (University of Texas MD Anderson Cancer Center, Houston, TX). The two lymphoma cell line models J45.01 and Toledo were purchased from the American Type Culture Collection (Manassas, VA). Blood samples from patients with leukemia or lymphoma were collected after obtaining written informed consent. This study was performed according to a protocol approved by the Institutional Review Board of the University of Texas MD Anderson Cancer Center, in accordance with the Declaration of Helsinki. Mononuclear cells were isolated using lymphocyte separation medium (Mediatech, Manassas, VA). All cell cultures were performed as previously described [13].

The PARP inhibitor niraparib, and the HDAC inhibitors romidepsin and panobinostat were obtained from Selleck Chemicals (Houston, TX). Decitabine, busulfan and melphalan were purchased from Sigma-Aldrich (St. Louis, MO). Stock solutions of all drugs were prepared in dimethyl sulfoxide immediately prior to use in the respective experiment.

### Cell proliferation assay

Cell suspensions were aliquoted (100 μl of 5 × 10^5^ cells/ml) into 96-well plates in the presence of drugs or solvent alone and continuously incubated at 37°C for 48 hours. The cells were analyzed for proliferation by the 3-(4,5-dimethylthiazol-2-yl)-2,5-diphenyl tetrazolium bromide (MTT) assay [41]. Graphical analyses including calculations of IC_20_ values were done using Prism 5 (GraphPad Software, San Diego, CA, USA). Drug combination effects were estimated based on the combination index (CI) values [42] calculated using the CalcuSyn software (Biosoft, Ferguson, MO, USA). This program was developed based on the median-effect method: CI < 1 indicates synergy, CI ≈ 1 is additive, and CI > 1 suggests antagonism.

### Apoptosis assay

Cell death by apoptosis following a 48-hour drug exposure was determined by flow cytometric measurements of phosphatidylserine externalization [43] with the Annexin-V-FLUOS (Roche Diagnostics, Indianapolis, IN) and 7-aminoactinomycin D (BD Biosciences, San Jose, CA) kits using a Muse Cell Analyzer (EMD Millipore, Billerica, MA). The extent of cleavage of poly(ADP-ribose) polymerase (PARP)-1 and CASPASE 3, determined by Western blot, was also used as an indicator of apoptosis.

### Western blot analysis

Cells (5 × 10^5^/ml) were exposed to drugs or solvent for 48 hrs, collected by centrifugation, washed with cold PBS, and lysed with cell lysis buffer (Cell Signaling Technology, Danvers, MA). The protein concentrations were determined using a BCA Protein Assay kit (Thermo Fisher Scientific, Rockford, IL). Proteins were resolved on polyacrylamide-SDS gels and blotted onto nitrocellulose membranes (Bio-Rad, Hercules, CA). Western blot analyses were done by chemiluminescence using the Immobilon Western Chemiluminescent HRP Substrate (EMD Millipore). The antibodies, their sources, and other relevant information are shown in Supplementary Table 2. X-ray films were scanned with the EPSON Perfection V750 PRO and analyzed with UN-SCAN-IT software (Silk Scientific, Orem, UT).

### Analysis of ROS production and mitochondrial membrane potential

Cells exposed to drug(s) for 48 hrs were analyzed for production of reactive oxygen species (ROS) using CM-H2DCFDA (5-(and-6)-chloromethyl-2′,7′-dichlorodihydrofluorescein diacetate, acetyl ester), an ROS indicator that diffuses into cells where it is oxidized to a fluorescent product (Life Technologies, Grand Island, NY). Briefly, cells were aliquoted (0.5 ml) into 5 ml tubes and 1 μl of 1.5 mM CM-H2DCFDA (freshly dissolved in dimethyl sulfoxide) was added. Cells were incubated at 37°C for 1 hr and immediately analyzed with a Gallios Flow Cytometer (Beckman Coulter, Inc., Brea, CA) using excitation/emission wavelengths of 492/520 nm. Geometric means of the fluorescence intensities were used in the analysis.

Changes in the mitochondrial membrane potential were measured using a JC-1 (5,5′,6,6′-tetrachloro-1,1′,3,3′-tetraethylbenzimidazolylcarbo cyanine iodide) mitochondrial membrane potential detection kit (Cayman Chemical, Ann Arbor, MI). Cells were exposed to drugs for 48 hrs and 0.5-ml cell suspension was aliquoted into 5-ml tubes. Diluted (1:10 with cell growth medium, 40 μl) mitochondrial membrane potential-sensitive fluorescent dye JC-1 reagent was added to each tube, incubated at 37°C for 20 min, and immediately analyzed by flow cytometry using the 530-nm (FL-1 channel, green) and 585-nm (FL-2 channel, red) band-pass filters simultaneously. Healthy cells with functional mitochondria and high membrane potential exhibit red fluorescence (aggregated JC-1), whereas dying cells with low membrane potential show green fluorescence (monomeric JC-1). The ratio of monomer/aggregate JC-1 was calculated.

### Isolation of soluble and chromatin-bound nuclear extracts

J45.01 cells were exposed to drugs for 48 hrs, collected by centrifugation, and washed with cold PBS. Soluble and chromatin-bound nuclear extracts were prepared using the Subcellular Protein Fractionation Kit for Cultured Cells (Pierce Biotechnology, Rockford, IL). Protein concentrations were determined and Western blot analysis was performed as described above.

### Immunoprecipitation assay

Nuclear extracts from J45.01 cells exposed to drugs were prepared using the NE-PER Nuclear and Cytoplasmic Extraction Reagents kit (Pierce Biotechnology). Approximately 400 μg total protein was diluted with cold PBS containing protease inhibitors (Roche Applied Science, Indianapolis, IN) to 500 μl and mixed with 50 μl (50% slurry) of pre-washed Pierce Protein A/G agarose beads (ThermoFisher Scientific, Inc.). The mixture was tumbled for 10 min at 4 °C and centrifuged at 14 000 × g for 10 min to eliminate non-specific binding species. The supernatant was mixed with 50 ng anti-acetylated lysine antibody (Cell Signaling Technology, Danvers, MA) and tumbled overnight at 4 °C. The mixture was centrifuged at 14 000 × g for 10 min and the supernatant was mixed with 50 μl (50% slurry) of pre-washed Pierce Protein A/G agarose beads, tumbled for 2 hrs at 4°C, centrifuged again, and the beads were washed two times before boiling in gel loading buffer (Cell Signaling Technology). Immunoprecipitated proteins were analyzed by Western blotting as described above.

### Statistical analysis

Results are presented as the average ± standard deviation of at least three independent experiments and statistical analysis was performed using a Student’s paired *t*-test with a two-tailed distribution.

## SUPPLEMENTARY MATERIALS TABLES


