# Supplementary material for: Combination of a hypomethylating agent and inhibitors of PARP and HDAC traps PARP1 and DNMT1 to chromatin, acetylates DNA repair proteins, down-regulates NuRD and induces apoptosis in human leukemia and lymphoma cells
Source: Oncotarget. 2017 Dec 17;9(3):3908–21. doi: 10.18632/oncotarget.23386 (PMC5790510; doi:10.18632/oncotarget.23386)
Supplement: Supplementary file 1 [file oncotarget-09-3908-s001.pdf]

Combination of a hypomethylating agent and inhibitors of PARP and HDAC traps PARP1 and DNMT1 to chromatin, acetylates DNA repair proteins, down-regulates NuRD and induces apoptosis in human leukemia and lymphoma cells

SUPPLEMENTARY MATERIALS

Supplementary Table 1: Identified acetylated lysine residues searched from the post-translational modification database (PHOSIDA)*

Protein	Process involved	Peptide Sequence	Lys position
Ku70/XRCC6	DSB repair via NHEJ	LEASGDYKYSGRDSL	30
		SRQIILEKEETEELK	330
		KEETEELKRFDDPGL	337
		ATPEQVGKMKAIVEK	460
		KMKAIVEKLRFTYRS	467
Ku80/XRCC5	DSB repair via NHEJ	DLSSRFSKSQLDIII	143
		HGPSFPLKGITEQQK	194
		SIRIAAYKSILQERV	264
		KVDEEQMKYKSEGKC	331
		MKYKSEGKCFSVLGF	337
		KSQIPLSKIKTLFPL	531
		QRFNNFLKALQEKVE	659
		FLKALQEKVEIKQLN	664
PARP1	ADP-ribosylation, Base-excision repair	EAGGVTGKGQDGIGS	96
		GQDGIGSKAEKTLGD	104
		TCKGCMEKIEKGQVR	130
		GTVIGSNKLEQMPSK	599
		FMKLYEEKTGNAWHS	620
DDB1	Nucleotide excision repair	DRDNKELKAFNIRLE	153
		KVIKSVGKIEHSFWR	1067
CHD4	NuRD component	RLKNNQSKFFRVLNG	884
		PMQKKYYKYILTRNF	987
		KGAADVEKVEEKSAI	1643
Histone 3	nucleosome assembly	RTKQTARKSTGGKAP	9
		ARKSTGGKAPRKQLA	14
		TGGKAPRKQLATKAA	18
		PRKQLATKAARKSAP	23
		LATKAARKSAPSTGG	27
		APSTGGVKKPHRYRP	36
		REIRRYQKSTELLIR	56
		REIAQDFKTDLRFQS	79
		KRVTIMPKDIQLARR	122

* 141.61.102.18/phosida/index.aspx.

Supplementary Table 2: List of primary antibodies, their sources and dilutions

Antigen	Source/Cat. #	Clone type*	Dilution**
β-ACTIN	Sigma/A5316	mAb	6000
ATM	Santa Cruz Biotech/25921	mAb	750
P-ATM (S1981)	Rockland/200-301-400	mAb	2000
CHD3	Cell Signaling/4241	pAb	3000
CHD4	Cell Signaling/11912	mAb-R	3000
CHK2	Cell Signaling/2662	pAb	2500
P-CHK2 (S19)	Cell Signaling/2666	pAb	2500
Cleaved CASPASE 3	Cell Signaling/9661	pAb	2500
DDB1	GeneTex/100130	pAb	3000
DNMT1	Santa Cruz Biotech/10222	pAb	700
HDAC1	Cell Signaling/5356	mAb	2500
HDAC2	Cell Signaling/5113	mAb	2500
Ac-Histone 3 Lys4	Active Motif/39381	pAb	2500
γ-Histone 2AX	EMD Millipore/05-636	mAb	3000
Histone 3	Abcam//1791	pAb	3000
Ku70	Own Laboratory	mAb	500
Ku80	Cell Signaling/2180	mAb-R	2500
MBD3	Cell Signaling/14540	pAb	3000
MDR1/ABCB1	Abcam/170904	mAb	2000
MRP1/ABCC1	Abcam/24102	mAb	2000
MTA1	Cell Signaling/5647	mAb-R	2500
NBS1	Cell Signaling/3002	pAb	2500
Nucleoporin	Santa Cruz Biotech/48373	mAb	700
PAR	Trevigen/#4336-BPC-100	pAb	3500
PARP1	Santa Cruz Biotech/8007	mAb	1000
RAD50	Cell Signaling/3427	pAb	2500
RBAP46	Cell Signaling/6882	pAb	2500

*pAb: polyclonal antibody; used anti-rabbit IgG for secondary antibody from Bio-Rad Lab.
mAb: monoclonal antibody; used anti-mouse IgG for secondary antibody from Bio-Rad Lab.
mAb-R: rabbit monoclonal antibody.
**Fold dilution in PBS with 0.05% Tween 20.
